# The Safety and Efficacy of Ultrasound-Accelerated Catheter-Directed Thrombolysis in Patients with Intermediate–High-Risk Pulmonary Embolism: Bo-NE-Experience

**DOI:** 10.3390/jcm12103459

**Published:** 2023-05-14

**Authors:** Hani Al-Terki, Andreas Mügge, Michael Gotzmann, Vedat Tiyerili, Friederike Klein, Marcus Franz, Sven Möbius-Winkler, Abdelrahman Elhakim

**Affiliations:** 1 Cardiology and Rhythmology Department, University Hospital St. Josef-Hospital Bochum, Ruhr University Bochum, Gudrunstraße 56, 44791 Bochum, Germany; andreas.muegge@ruhr-uni-bochum.de (A.M.);; 2 Cardiology and Rhythmology, St.-Johannes Hospital Dortmund, 44137 Dortmund, Germany; 3 Department of Internal Medicine I, Division of Cardiology, University Hospital Jena, 07747 Jena, Germany; 4 Cardiology Department, Schoen Hospital, 23730 Neustadt in Holstein, Germany

**Keywords:** EKOS^TM^, pulmonary embolism, actilyse, USAT

## Abstract

Ultrasound-accelerated thrombolysis (USAT) is an advanced interventional therapy for patients with intermediate–high-risk pulmonary embolism (PE) who deteriorated on anticoagulation or for high-risk patients for whom systemic thrombolysis is contraindicated. The aim of this study is to investigate the safety and efficacy of this therapy with a focus on the improvement of vital signs and laboratory parameters. Seventy-nine patients with intermediate–high-risk PE were treated with USAT from August 2020 to November 2022. The therapy significantly decreased the mean RV/LV ratio from 1.2 ± 0.22 to 0.9 ± 0.2 (*p* < 0.001) as well as the mean PAPs from 48.6 ± 11 to 30.1 ± 9.0 mmHg (*p* < 0.001). The respiratory and heart rate decreased significantly (*p* < 0.001). Serum creatinine decreased significantly from 1.0 ± 0.35 to 0.9 ± 0.3 (*p* < 0.001). There were 12 access-associated complications, which could be treated conservatively. One patient had haemothorax after the therapy and had to be operated on. USAT is an effective therapy for patients with intermediate–high-risk PE, with favourable hemodynamic, clinical, and laboratory outcomes.

## 1. Introduction

Pulmonary embolism (PE) is the third leading cause of cardiovascular mortality [1]. The technological landscape for the management of acute intermediate–high- and high-risk PE is rapidly evolving. Patients with intermediate–high-risk PE, even if normotensive, are at high risk of in-hospital and latent mortality [1].

The guidelines of the European Society of Cardiology (ESC) recommend rescue reperfusion therapy for patients with acute intermediate–high-risk PE who are deteriorating on anticoagulation. Alternatively, catheter-directed therapies, such as ultrasound-accelerated thrombolysis (USAT), should be considered [2].

USAT using EKOS^TM^ (Boston Scientific, Marlborough, MA) is an approach combining low-power ultrasound energy with a low-dose thrombolytic agent to achieve clot dissolution. Ultrasound uses cavitation-induced microstreaming, which loosens the fibrin strands within the clot, increasing its surface area. In addition, the ultrasound enhances the transport of the thrombolytic agent across the clot and makes it more permeable [3,4].

In patients with acute intermediate–high-risk PE who deteriorated on anticoagulation or systemic thrombolysis was contraindicated, USAT has been already used therapeutically. In these studies, the ratio between the right ventricle (RV) and the left ventricle (LV), the systolic pulmonary artery pressure (PAP), and the RV function at 24 h [5,6,7,8] improved significantly. In all of those cases, different dosages of alteplase (rt-PA) were used.

The OPTALYSE study aimed to determine the lowest optimal (rt-PA) dose and delivery duration using USAT for the treatment of acute, intermediate–high-risk PE. The patients were randomized into four groups and received different rt-PA dosages. Patients who were included in the third arm received 6 mg/lung/6 h and showed favourable improvement in the RV-to-LV diameter ratio, with low major bleeding rates. However, the sample size of 28 patients was too small. We present data from our two-centre experience with EKOS^TM^ in seventy-nine PE patients who received the same dosage and the same delivery duration accordingly to the third therapeutic arm of the OPTALYSE study. Our Study focuses on the effect of the therapy on vital signs and laboratory values.

## 2. Methods

Between August 2020 and November 2022, seventy-nine patients with intermediate–high-risk PE received USAT using the EKOS^TM^ Endovascular System at Josef Hospital Bochum or Schoen Clinic Neustadt in Holstein in Germany and were included in this retrospective registry study.

The diagnosis in all patients was confirmed by contrast-enhanced computed tomography. The RV function/strain was evaluated using transthoracic echocardiography (TTE) and by measuring cardiac biomarkers (troponin T and NTproBNP). All PE patients were classified according to the ESC guidelines as low, intermediate–low-, intermediate–high- or high-risk PE. Patients with right heart dysfunction, elevated Troponin and NTproBNP, and an elevated simplified pulmonary embolism severity index (sPESI) ≥ 1 are classified as intermediate–high risk and were included in this study.

Inclusion criteria were ages ≥ 18 years, an RV/LV ratio ≥ 1, and hemodynamic deterioration on anticoagulation. The deterioration was defined as increasing tachycardia or a worsening of systolic blood pressure by at least 20 mmHg under the initiated therapy with Heparin. These subjects were estimated as suitable candidates for USAT with EKOS^TM^, which was performed in the cardiac catheterization laboratory in the first 24 h after admission.

The exclusion criteria were active bleeding, a history of intracranial or intraocular bleeding at any time, stroke or transient ischemic attack within the past 6 months, central nervous system neoplasm or metastatic cancer, platelet count < 100,000/mcl, and patients with a life expectancy less than 6 months.

The EKOS^TM^ system involves positioning one (for unilateral PE) or two pulmonary arterial infusion catheters, one into each main pulmonary artery (PA), under fluoroscopic guidance via percutaneous transvenous access. The procedure was performed with continuous haemodynamic and electrocardiographic (ECG) monitoring. For 6 h, 6 mg of rt-PA was continuously administered per catheter, with a rate of 1 mg/h. Heparin was administered concomitantly, with doses being determined using a hospital-defined nomogram that protocolizes dosing to target partial thromboplastin of 60 s. After 6 h, the system was stopped, and the infusion catheters were removed, followed by compression of the femoral vein for 4 h.

For an additional 2 h, the intravenous therapy with unfractionated heparin was continued, followed by oral anticoagulants.

Twenty-four hours after EKOS therapy and before discharge, another TTE was conducted to measure the RV function (TAPSE: tricuspid annular plane systolic excursion), PAPs, RV end-diastolic diameter (RVEDd), and the RV/LV ratio. In addition, creatinine, troponin T, and NTproBNP were monitored 24 h after the therapy. Vital parameters (respiratory rate, heart rate, and blood pressure) were regularly monitored from admission to transfer to the normal ward.

**Figure 1:**
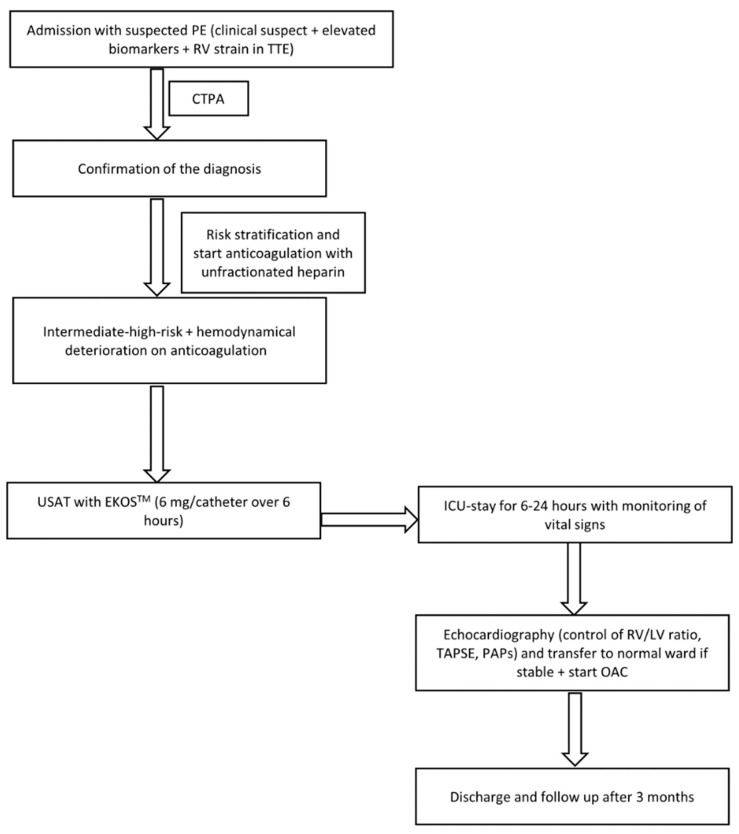
Study flow chart.

For midterm evaluation, patients were followed up for at least 3 months in the outpatient department. A physician interviewed all patients. Right ventricular function was evaluated using TTE (Figure 1 shows the study flow chart)

### Statistical Analysis

Continuous variables were expressed as mean ± standard deviation, and the categorical variables were expressed as percentages. All variables were normally distributed. The statistical analyses were performed using the SPSS Version 25.0 for Windows. Comparison between the variables was performed using the 2-tailed *t*-test. A *p*-value < 0.05 was considered statistically relevant. All statistical analyses were performed by a blinded analyst.

## 3. Results

### 3.1. Study Population

Seventy-nine patients with intermediate–high-risk PE who underwent USAT with EKOS^TM^ were included in this registry and retrospectively analysed. Forty-one of them were women (51.9%). The mean age was 66.7 years (SD: 13.8), and the mean body mass index (BMI) was 29 kg/m^2^ (SD: 6.4). Eight patients (10.1%) had chronic heart disease, 40 (50.6%) had arterial hypertension, and seven (8.9%) had diabetes mellitus. Fourteen (17.7%) had been previously diagnosed with PE. In twenty-seven subjects (34.2%), the PE was triggered by cancer (*n* = 14, 17.7%) or immobility (*n* = 13, 16.5%). Baseline characteristics are summarised in Table 1.

### 3.2. Initial Presentation, Clinical and Echocardiographic Parameters at Admission

The most common primary complaint was dyspnea (63 patients, 79.7%). Seven patients (8.9%) suffered from syncope and five (6.3%) presented after cardiopulmonary resuscitation (CPR), of whom three patients had post-operative PE, one patient had gastrointestinal bleeding two weeks ago, and one patient was under oral anticoagulation with apixaban because of atrial fibrillation. Thus, intravenous systemic thrombolysis was contraindicated. Two patients (2.5%) presented with angina pectoris, one (1.3%) with dizziness as the main complaint, and one (1.3%) presented with cough.

The mean heart rate at admission was 96.7 bpm (SD: 16), the mean systolic blood pressure was 137 mmHg (SD: 25), and the mean respiratory rate was 20 per minute (SD: 5).

The initial RV/LV ratio measured by TTE was 1.24 (SD: 0.22). The mean RV/LV ratio measured on CT was 1.3 (SD: 0.35). The mean PAP was 48.6 mmHg (SD 11). The TAPSE was 19.4 mm (SD: 6).

The initial mean creatinine value was 1.0 (SD: 0.35), the initial mean NTproBNP was 4278.1 pg/mL (SD: 6520) (normal value < 125 pg/mL), and the mean haemoglobin value was 12.9 mg/dL (SD: 2.2). Table 2 summarizes the clinical, laboratory, and echocardiographic parameters at admission.

### 3.3. Procedural Data

Five patients (6.3%) had a right-sided PE and were treated with just one EKOS^TM^ catheter in the right pulmonary artery. Four patients (5.2%) had a left-sided PE. The majority of patients (*n* = 70 patients, 88.5%) showed a bilateral PE. The mean procedural time was 13.3 min (SD: 9.4), and the mean fluoroscopy time was 10.9 min (SD: 6.9). Table 3 summarizes the procedural data and the post-USAT stay.

### 3.4. Post-Procedural Data (Clinical Parameters, Echocardiography, and Laboratory)

The RV/LV ratio, PAP, and TAPSE decreased significantly after USAT, as well as the NTproBNP and creatinine. Table 4 summarizes the mean values before and after USAT. All control values were measured at 24 h after the lysis.

### 3.5. Complications

There was no device failure in our study. Three in-hospital deaths (3.8%) occurred. One patient died as a result of an air embolism during an operation that took place three weeks after the USAT therapy, and the other two patients died due to pneumonia-associated septic shock. There were no device-related deaths. Seven subjects (8.9%) had mild access-site hematoma, and three patients (3.8%) showed a pseudoaneurysm due to the mis-puncture of the arteria femoralis. All access-related complications were treated conservatively without the need for surgical intervention and without blood transfusion. One patient had haemothorax with severe bleeding, according to the GUSTO bleeding score. The patient underwent surgery after the transfusion of one blood unit.

### 3.6. Follow-Up after 3 Months

**Figure 2:**
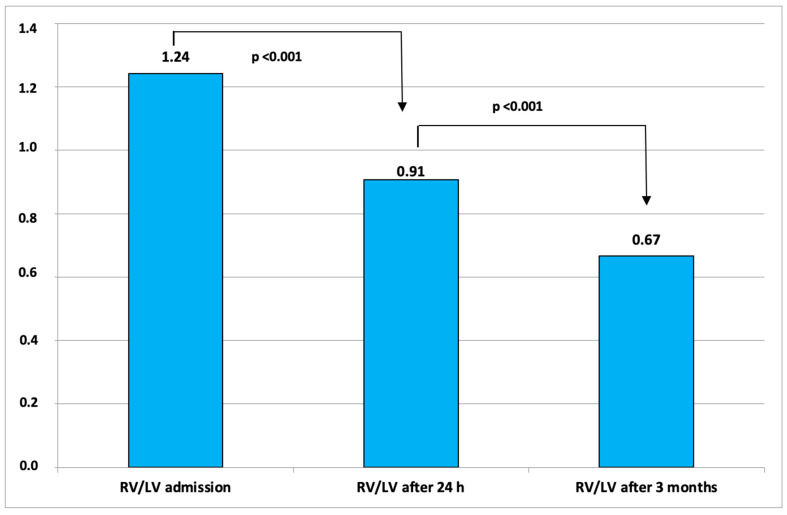
Changes of RV/LV ratio from baseline to 3 months after therapy.

Our patients underwent a three-month post-interventional evaluation in our outpatient clinic. Three patients died during the follow-up due to advanced cancer. No major bleeding, according to the GUSTO score, occurred. Additionally, there were no recurrent PEs. The follow-up echocardiographic parameters are listed in Table 5 below. Figure 2 shows the changes of RV/LV ratio from baseline to 3 months after therapy.

## 4. Discussion

Our study shows that USAT with EKOS^TM^ with 1 mg/h per catheter for 6 h is a safe and efficient therapeutic modality for patients with intermediate–high-risk PE, with a positive effect concerning not only the echocardiographic parameters but also the clinical parameters and the laboratory values.

PE is the third leading cause of cardiovascular mortality [1]. The PE therapy is often limited to anticoagulation. However, patients with an intermediate–high-risk PE have a high mortality even if they are normotensive [1]. In such cases, catheter-based techniques are of interest because of the increased risk of major bleeding or stroke associated with systemic thrombolysis and the theoretical limitation of systemic-infused thrombolytic agents; the latter may cause blood to be shunted toward the unobstructed pulmonary arteries rather than those with obstruction [6,9]. However, evidence concerning USAT is still considerably less robust than that for systemic thrombolysis.

The OPTALYSE study aimed to determine the lowest optimal (rt-PA) dose and delivery duration using USAT for the treatment of acute, intermediate–high-risk PE. The patients were randomized into four groups. The rt-PA dose ranged from 4 to 12 mg per lung, and the infusion duration ranged from 2 to 6 h; the first arm received (4 mg/lung/2 h), the second arm received 4 mg/lung/4 h), the third arm received 6 mg/lung/6 h, and the fourth arm received 12 mg/lung/6 h. Similarly, to our collective, the third group received 6 mg per lung for 6 h. This dose was associated with an improvement in the RV/LV ratio. However, the sample size of 28 patients in this group was too small to draw any firm conclusions [7].

We present our registry data of seventy-nine patients of intermediate–high-risk PE patients who underwent USAT with EKOS^TM^ at two high-volume centres in Germany with the same dose and the same delivery duration of the third arm of OPTALYSE. Our results demonstrate that USAT significantly reduces pulmonary artery pressure overload and improves RV function with relatively low rates of complications. Our study investigates the benefits of USAT on clinical parameters, such as heart rate and respiratory rate, which were also significantly reduced 24 h after therapy.

### 4.1. Procedural Data, ICU and Total Hospital Stay

EKOS^TM^ had a high rate of device success in our study. This was defined as the successful delivery of the catheter into the pulmonary arteries without complications, combined with a decrease in the RV/LV ratio after therapy to <1.0. This was achieved in all patients (100% success rate). The procedural time was 13.3 ± 9.4 min, the fluoroscopy dose was 1518 ± 1111 cGy/m^2^, and the fluoroscopy time was 10.9 ± 6.9 min. In the majority of the patients (*n* = 67, 84% of the population), no contrast agent was necessary. Unfortunately, we could not compare these findings with prior publications, as they were not communicated in other publications (ULTIMA, SEATTLE-II, Jena-Experience, SUNSET, OPTALYSE or Kaymaz et al.) [5,7,8,10,11,12]. However, we think that these data are of immense importance, especially if a comparison between USAT and another interventional technology is performed in the future to define which patients would derive the greatest net benefit from their use in various clinical settings.

The mean ICU stay of the entire cohort in our study was 1.7, similar to the SUNSET Trial (2 days). However, the total median hospital stay was 13 days longer than SUNSET (7.7 days). This could be explained by the higher percentage of complications in our study compared to this reported in SUNSET.

### 4.2. The Echocardiographic Data

Regarding the RV/LV ratio, PAPs, and TAPSE, our findings are similar to those shown by ULTIMA [5], OPTALYSE [7], SEATTLE-2 [8], Jena Experience [10], Kaymaz et al. [11], and SUNSET Trial [12].

In the ULTIMA and SEATTLE-2 trials, the RV/LV ratio after EKOS^TM^ decreased by 0.3 and 0.42, respectively, similar to the results of Kaymaz et al. and Klein et al. (0.27 and 0.24, respectively) [10]. The mean decrease in the RV/LV ratio 48 h after the therapy in the third arm of the OPTALYSE study was 0.42 ± 0.32. In our study, the mean decrease in RV/LV ratio from baseline to 24 h after the therapy was 0.33. In contrast to our study, SEATTLE-II, SUNSET, and OPTALYSE measured the echocardiographic parameters 48 h after the therapy.

The decrease in the PAPs after the therapy in the ULTIMA and SEATTLE-II trials was 9.8 mmHg and 14.3 mmHg, respectively. In our study population, this was slightly higher, at 18.42 mmHg, although very close to the result of Kaymaz et al. (17.8 mmHg). The mean change in PAPs in the third arm of the OPTALYSE study was 12.5 ± 11.0 mmHg. Compared to the studies mentioned above, we showed a greater drop in the PAPs with the use of a lower dosage of thrombolytic agents (6 mg rtPA per catheter in our study compared to 11.5 mg in the Jena-Experience trial). This could be explained by the fact that more than 80% of the patients included in the Jena-Experience trial received just one catheter (in total 11.5 mg rt-PA), whereas 88.5% of our patients received two catheters with a total dose of 12 mg rt-PA.

As in the ULTIMA trial and the study by Kaymaz et al., there was a significant increase in TAPSE after the therapy (4.4 mm and 3 mm, respectively). In our study population, EKOS^TM^ could improve TAPSE significantly by 4.7 mm.

### 4.3. The Echocardiographic Data 3 Months after Therapy

Regarding the midterm outcomes, our data are comparable to those achieved in the aforementioned studies. The mean decrease in the RV/LV ratio from baseline to follow-up in the ULTIMA and Jena-Experience trials was 0.35 and 0.41, respectively, compared to 0.56 in our study population. The mean reduction in PAPs in the ULTIMA and Jena-Experience trials was 12.3 mmHg and 22 mmHg, respectively, compared to 20.5 mmHg in our study. No device-related deaths occurred during follow-up. The OPTALYSE study does not mention these parameters after 3 months.

### 4.4. Vital Signs after Lysis

Our study is the first to focus on the effect of USAT on vital signs. We showed that USAT significantly decreased the respiratory and heart rates of patients within 24 h. The systolic blood pressure could not be improved. This is mainly due to the fact that patients who were treated with USAT in our study were classified as intermediate–high-risk, which means that they are still normotensive. Hypotensive PE patients are at high risk and were not included in this study.

### 4.5. Laboratory Parameters after the Therapy (Creatinine and NTproBNP)

Renal insufficiency and acute kidney injury (AKI) due to acute PE may have a significant impact on early negative prognosis [13]. Acute PE causes elevated pressure in the pulmonary artery, which in turn could cause a right heart insufficiency and then renal insufficiency. Theoretically, each therapy which leads to a drop in the pulmonary artery pressure would improve renal function. In our study, creatinine values could be significantly decreased after lysis. Whether this is due to EKOS^TM^ or due to the fluids, which are given during USAT therapy, is not clear.

NTproBNP is a predictor of adverse short-term clinical outcomes in patients with acute pulmonary embolism [14]. In accordance with the findings of the Jena-Experience trial, in our population, EKOS^TM^ significantly reduced the levels of NTproBNP, suggesting that this therapy could positively affect the short-term prognosis of patients.

### 4.6. In-Hospital Mortality

The in-hospital mortality rate in our population was 3.8%. One patient died two weeks later due to an air embolism during orthopaedic surgery, and two died due to pneumonia-related septic shock. The mortality in our study is slightly higher compared to that of the SEATTLE-II study (2.7%), SUNSET (2.5%), ULTIMA trial (0%), and the third arm of OPTALYSE (0%) but almost identical to that of the Jena-Experience trial (3.9%) and slightly lower than that of the study by Kaymaz et al. (5.7%). This could be explained by the differences in the comorbidities of the different study populations. For example, the ULTIMA trial excluded post-operative PEs and patients above 80, which was not the case in our cohort. The SEATTLE-II study also excluded post-operative PEs, which could have affected the in-hospital mortality of the investigated population.

### 4.7. Complications

All of the access-associated complications were conservatively managed without the need for surgical interventions or blood transfusions. One patient had haemothorax with severe bleeding, according to the GUSTO bleeding score. This could be due to lysis or due to the manipulation during the insertion of the EKOS catheter.

Of note, since no bleeding scale has been validated in PE patients receiving thrombolytics, the criteria for minor and major bleeding differ between the studies. In the SEATTLE II trial and in our study, the GUSTO bleeding criteria were used, whereas the researchers in the Jena-Experience trial used the BARC (Bleeding Academic Research Consortium) score. Both scores have been validated for acute coronary syndrome but not for PE.

The rate of severe complications in our study was quite low (1.2%). We also used a lower dosage of rt-PA in our study compared to those of the ULTIMA, SEATLLE-II, and Kaymaz et al. The rate of access-associated complications was 12.9%. This is higher than in SEATTLE II (10%), SUNSET (7.5%), ULTIMA (0%), and Kaymaz et al. (7%) but comparable to the Jena-Experience (13.7%). This may be due to the fact that the venous puncture was not ultrasound-guided in all patients. Our study shows that USAT is effective and safe without the need for a high rt-PA dosage or a long treatment time. Patients who were included in the third arm of the OPTALYSE study received a 6 mg rt-PA per catheter for 6 h. However, one patient developed anaemia in the setting of a bleeding uterine fibroid after receiving an additional 12 mg rt-PA beyond the assigned 12 mg. The first arm of the study (4 mg rt-PA over 2 h) does not have any major bleeding events. In the second arm (4 mg rt-PA over 4 h), one patient (3.7%) developed anaemia and intracranial haemorrhage. The fourth arm (12 mg rt-PA over 6 h) had two major bleeding events (11%), intracranial bleeding and bleeding from a splenic pseudoaneurysm.

## 5. Limitations

In this study, we present our experience regarding the use of USAT in two high-volume centres in Germany, with both in-hospital and short-term outcomes. We have introduced new aspects of the benefits of USAT, such as the post-therapeutic improvement of vital signs and creatinine levels. The main limitation is that this is a retrospective study lacking a control group and randomisation.

Large, randomised studies are still needed to validate our new findings regarding the heart and breathing rate reduction as well as the decrease in creatinine and to determine the optimum rtPTA dosage needed to achieve the best outcomes.

## 6. Conclusions

USAT is an effective and safe treatment for patients with intermediate–high-risk PE and can improve clinical and echocardiographic parameters. The use of 6 mg rt-PA/catheter for 6 hr (similar to the third arm of the OPTALYSE study) correlates with a low rate of complications. Further studies concerning the necessary dosage of the thrombolytic agent and treatment duration, as well as long-term outcomes, are still needed.

## Figures and Tables

**Table 1:** Baseline characteristics.

	Patient Characteristics	N = 79
**Characteristics**	Age, mean (SD)	66.7 (13.8)
	females	41 (51.9%)
	BMI, mean (SD)	29 (6.4)
**Comorbidities**	Arterial Hypertension, *n* (%)	40 (50.6%)
	Diabetes mellitus, *n* (%)	7 (8.9%)
	CHD, *n* (%)	8 (10.1%)
	Coronary artery disease, *n* (%)	5 (6.3%)
	Cancer, *n* (%)	14 (17.7%)
	Previous PE, *n* (%)	14 (17.7%)
	Immobility, *n* (%)	13 (16.5%)
	COPD, *n* (%)	3 (3.8%)
	Asthma, *n* (%)	1 (1.3%)
	COVID, *n* (%)	1 (1.3%)
	Lung emphysema, *n* (%)	1 (1.3%)
	OSAS, *n* (%)	1 (1.3%)

BMI: Body mass index, OSAS: Obstructive sleep apnea syndrome, COPD: chronic obstructive pulmonary disease, CHD: chronic heart failure, SD: standard deviation.

**Table 2:** Clinical, chemical laboratory, and echocardiographic parameters at admission.

	Patient Characteristics	N = 79
**Initial presentation**	Dyspnea, *n* (%)	63 (79.7%)
	Syncope, *n* (%)	7 (8.9%)
	CPR, *n* (%)	5 (6.3%)
	Angina pectoris, *n* (%)	2 (2.5%)
	Dizziness	1 (1.3%)
	Cough, *n* (%)	1 (1.3%)
**Clinical parameters at admission**	Heart rate, mean (SD)	96.7 bpm (16)
	Systolic pressure, mean (SD)	137 mmHg (25)
	Respiratory rate, mean (SD)	20 (5)
	Oxygen saturation, mean (SD)	90.7% (7.8)
**Echocardiographic parameters at admission**	RVEDD, mean (SD)	45.7 mm (5.5)
	LVEDD, mean (SD)	24 mm (16)
	PAPs, mean (SD)	48.6 mmHg (11)
	TAPSE, mean (SD)	17 mm (5)
	RV/LV ratio, mean (SD)	1.24 (0.22)
**Laboratory-chemical values**	Creatinine, mean (SD)	1.0 mg/dL (0.35)
	NTproBNP, mean (SD)	4278.1 pg/mL (6520)
	Troponin T, mean (SD)	0.20 pg/mL (0.36)
	Hemoglobin, mean (SD)	12.9 mg/dL (2.2)

CPR: Cardiopulmonary resuscitation, RVEDD: right ventricle end-diastolic diameter, LVEDD: left ventricle end-diastolic diameter, PAP: pulmonary artery pressure, TAPSE: Tricuspid annular plane systolic excursion.

**Table 3:** The procedural data and post-interventional stay.

		N = 79
**Procedural data**	Procedural time (min), mean (SD)	13.3 (9.4)
	Contrast agent (ml), mean (SD)	4.8 (13.6)
	Fluoroscopy dose (Gy), mean (SD)	1518 cGY/cm^2^ (1111)
	Fluoroscopy time (min), mean (SD)	10.9 (6.9)
**Post-procedural stay**	ICU-Stay (days), mean (SD)	1.7 (4.6)
	Total-Stay (days), mean (SD)	13.3 (9.4)

ICU: Intensive Care Unit.

**Table 4:** The mean values before and after the therapy.

	Before USAT	After USAT	Mean Absolute Change (95% CI)	t	*p*-Value
Heart rate (bpm)	96.7 ± 16	82.4 ± 9.4	14.2 (11–17.6)	8.7	<0.001
Respiratory rate (/min)	20.5 ± 4.6	16.6 ± 8.2	3.9 (1.7–6.0)	3.6	<0.001
Systolic blood pressure (mmHg)	137 ± 25	134 ± 23	2.6 (−3.3–8.7)	0.88	0.3
TAPSE (mm)	17.05 ± 5	21.7 ± 3.9	4.7 (−5.7 to −3.6)	−8.9	<0.001
PAPs (mmHg)	48.6 ± 11	30.1 ± 9.0	18.42 (15.9–20.8)	15.2	<0.001
RVEDD (mm)	45.8 ± 5.6	37 ± 5.6	8.7 (7.6–9.9)	15.5	<0.001
RV/LV ratio	1.2 ± 0.22	0.9 ± 0.2	0.33 (0.27–0.39)	11.5	<0.001
NTproBNP (pg/mL)	4278 ± 6520	3373 ± 6069	904 (423–1484)	3.1	0.003
Creatinine (mg/dL)	1.0 ± 0.35	0.9 ± 0.3	0.12 (0.06–0.17)	4.3	<0.001
Hemoglobin (mg/dL)	12.94 ± 2.1	11.38 ± 2.0	1.56 (0.14–1.2)	10.9	<0.001

TAPSE: Tricuspid annular plane systolic excursion, PAP: systolic pulmonary artery pressure, RVEDD: right ventricle end-diastolic diameter.

**Table 5:** The follow-up data.

	After USAT-after 3 Months	t	*p*-Value	Δ from Baseline to 3 Months after Therapy	t	*p*-Value
TAPSE (mm)	−0.3 ± 4.1	−0.5	0.61	−4.5 ± 5.6	−5.3	<0.001
PAPs (mmHg)	−4.1 ± 6.7	4.3	<0.001	−20.5 ± 9.9	14.5	<0.001
RVEDD (mm)	−4.4 ± 7.5	3.9	<0.001	−12.8 ± 7.7	11.0	<0.001
LVEDD (mm)	3.4 ± 9.0	−2.7	0.009	3.6 ± 8.5	−12.7	<0.001
RV/LV ratio	−0.3 ± 0.33	6.7	<0.001	−0.56 ± 0.34	11.7	<0.001

TAPSE: Tricuspid annular plane systolic excursion, PAP: systolic pulmonary artery pressure, RVEDD: right ventricle end-diastolic diameter, LVEDD: left ventricle end-diastolic diameter, RV/LV ratio: right ventricle/left ventricle ratio.

## Data Availability

Data is unavailable due to privacy or ethical restriction.

## Abbreviations

Acute kidney injury	AKI
Alteplase	rt-PA
Body mass index	BMI
Cardiopulmonary resuscitation	CPR
Catheter-directed-thrombolysis	CDT
Chronic Heart disease	CHD
Chronic obstructive lung disease	COPD
Computed tomography	CT
Computed tomography pulmonary angiography	CTPA
Electrocardiogram	ECG
European society of cardiology	ESC
Left ventricle	LV
N-Terminal Pro-Brain Natriuretic Peptide	NTproBNP
Obstructive sleep apnoea syndrome	OSAS
Pulmonary embolism	PE
Right ventricle	RV
Standard deviation	SD
Systolic pulmonary artery pressure	PAPs
Transthoracic echocardiography	TTE
Tricuspid annular plane systolic excursion	TAPSE
Ultrasound-accelerated thrombolysis	USAT
